# Knowledge and ethical perception regarding organ donation among medical students

**DOI:** 10.1186/1472-6939-14-38

**Published:** 2013-09-27

**Authors:** Nisreen Feroz Ali, Amal Qureshi, Basmah Naser Jilani, Nosheen Zehra

**Affiliations:** 1 Department of Community Health Sciences, Ziauddin Medical University, ST-4/B, Block 6, Clifton, Karachi, Pakistan; 2 Fifth year medical students, Ziauddin Medical University, ST-16, Block B, North Nazimabad, Karachi, Pakistan

**Keywords:** Organ donation, Knowledge, Ethical perception, Transplantation, Transplantation of human organ and tissues bill 2007, Medical students, First years and fourth years, Willingness to donate

## Abstract

**Background:**

To determine the knowledge and ethical perception regarding organ donation amongst medical students in Karachi- Pakistan.

**Methods:**

Data of this cross sectional study was collected by self administered questionnaire from MBBS students of Ziauddin University from 2010 to 2011. Sample size of 158 (83 First years and 75 Fourth years) were selected by convenient sampling and those students who were present and gave consent were included in the study. The data was analyzed by SPSS version 20.

**Results:**

A total of 158 participants from Ziauddin Medical University filled out the questionnaire out of which 83(52.5%) were first years and 75(47.5%) were fourth year medical students. Mean age of sample was 20 ± 1.7. Majority of students were aware about organ donation with print and electronic media as the main source of information. 81.6% agreed that it was ethically correct to donate an organ. In the students’ opinion, most commonly donated organs and tissues were kidney, cornea, blood and platelet. Ideal candidates for donating organ were parents (81%). Regarding list of options for preference to receive an organ, most of the students agreed on young age group patients and persons with family. Willingness to donate was significantly associated with knowledge of allowance of organ donation in religion (P=0.000).

**Conclusion:**

Both 1st year and 4th year students are aware of Organ Donation, but there is a significant lack of knowledge regarding the topic.

## Background

The demand for organs for transplantation in Pakistan continues to overwhelmingly exceed the limited supply. Kidney transplant is the main organ transplant carried out in Pakistan, and only a handful of liver transplants have been carried out to date even though 10,000 people die due to liver failure each year [1]. An estimated 50,000 people die each year due to end stage organ failure, 18,000 people a year go into end stage renal failure of which only 10% receive dialysis and only 4-5% have the good fortune to be transplanted at a rate of 5 per million population [2,3]. Pakistan boasts of a population of 180 million people and is the sixth most populous country in the world [4]. In comparison to this staggering population there are only 148 dialysis centers, mostly in the private sector. Of the 25 transplant centers, nine are in the government sector and the rest in the private sector where a transplant cost is anywhere from $6000 to $10,000, which is unaffordable by the vast majority of the population [5].

In order to curtail illegal organ trade and associated exploitation of the impoverished and vulnerable the ‘Transplantation of Human Tissues and Organ Bill’ was passed unanimously by the Pakistan Parliament in 2007 [6]. The bill proposes a number of measures, including the restriction of organ donation only to close blood relatives. All donations have to be evaluated by a committee of medical experts to determine voluntariness and strict actions to be taken against those who disobey the law. The law also defined brain death and allowed deceased organ donation. It also created the “Human Organs Transplantation Authority” which established a transplant registry and recognized centers for transplantation. The implementation of cadaver legislation is essential to solve the problem of organ shortage and illegal organ trade.

In light of the current situation there was a dire need for a forum that encourages education and research in the field of organ donation; hence the “Transplantation Society of Pakistan” was formulated. Its mission includes promotion of the deceased donor program in Pakistan, along with seminars meetings and symposia to address medical, legal, ethical and social aspects of organ donation [7].

General public opinion surveys have found that while most people have a positive attitude towards organ donation and transplantation this seldom results in concrete action^.^ The public’s lack of action towards organ donation is consistently sited as the major factor for the current shortage of organs for transplantation [8]. This attitude can be a result of multifarious reasons, religious being one of the main. Many religions, though favorable towards the ideology of organ donation are hesitant about the criteria involved in this procedure. In a survey done on Muslims about their attitude towards donation 68.5% agreed to the idea of donation but only 39.3% believed it was compatible with Islam [9]. Another study showed that even though 88.2% of religious authorities allowed donation only 1.4% of them were willing to donate their organs [10]. Other issues include ethical grounds, political reasons, moral and cultural inhibitions. Also cases of tissue mismatch, recipient safety and organ conditions have created doubts in the minds of many about the actual significance of organ donation. In some systems, family members may give consent or refusal, or may even veto a potential donation even if the donor has consented. Asian countries such as China, Japan, Pakistan and India are amongst those where the knowledge and practice of organ donation is most lacking and where a diverse ethical perception is seen [11].

A research done to assess the attitude of postgraduate medical students towards organ donation showed that 89% of them wished to donate their organs [12]. If this level of knowledge and awareness is also imparted to the general public it would drastically enhance the number of people willing to donate. The best way to impart this knowledge is through doctors and thus it is essential to assess their attitude towards this topic. A survey [13] showed that the information required about organ donation was higher in the visitors that came to hospitals and so it is essential that doctors have the knowledge which can then be imparted appropriately. A local survey done in selected public areas of Karachi, Pakistan showed that amongst the general population 35.3% people expressed a high motivation to donate, and this was significantly associated with the level of education [14].

At present, our research comes as one of the few to analyze the level of awareness and ethical perception seen amongst medical students in Pakistan. With no formal course dedicated to organ transplantation at our institution, the results we found are intriguing and interesting. The objective was to acquire a general idea about the way medical students perceived organ donation and their knowledge regarding it and whether clinical exposure, which gradually increases in the five years of medical school, has any impact on it. Hence our target population was the first year medical students and final year medical students.

## Methods

The study was conducted to ascertain the knowledge and ethical perception regarding Organ Donation amongst medical students in Karachi. After approval from the ethical review committee, a cross sectional survey was conducted at Ziauddin University, during 2010-2011. With an average of ninety students in each year of study, the total medical student population at Ziauddin University is around 450 students. The class strength of first year was 105 and of fourth year were 90 students. Using convenient sampling, a total of 158 medical students, filled out the questionnaire, 83 students from first year and 75 students from fourth year. Participation in the study was voluntary. Inclusion criteria for the study population were students enrolled in first year and fourth year while the exclusion criteria was those who were not present and did not give consent. Pakistan has a five year MBBS program consisting of first two preclinical years and subsequent three clinical years. There is no formal course dedicated to the teaching of Organ Transplantation at the institution.

### Questionnaire

The Data was collected through a self administered questionnaire. The questionnaire was intended to analyze information in four categories which included, assessing the knowledge, individual perception, willingness to donate an organ and ethical beliefs about Organ Donation. Demographic information such as age, gender, religion and year of medical school was also included. Questionnaires were distributed in the classes of the respective years’ after a scheduled lecture. There were a total of 30 survey questions with a box checking format consisting of options which included, Yes, No and Don’t Know; while some were multiple response questions, consisting of, sources of awareness about Organ Donation, which organs can be donated, who are the eligible donors for donating an organ and what are the criteria’s’ for organ matching. The questionnaires were administered to the students with no prior information or announcements in order to minimize response bias. They were collected back immediately after anonymous completion.

### Statistical analysis

Data was entered and analyzed on Statistical Packages for the Social Sciences (SPSS) version 20 by IBM Corporation, America. All qualitative variables are described through frequencies and percentages and all quantitative variables are illustrated through mean and standard deviation. Chi- square test was applied and P values < 0.05 were considered significant.

## Results

A total of 158 participants from Ziauddin Medical University filled out the questionnaire out of which 83(52.5%) were first years and 75 (47.5%) were fourth year medical students. There were a total 105 students in first year and 90 students in fourth year. Response rate for first year was 79.04%, for fourth year was 83.3% and over all it is 81.02%. The mean age of the students was 20 ± 1.7 years. The sample included 58 (36.7%) males and 100 (63.3%) females, and almost all participants 154 (97.5%) were Muslims.

Virtually all participants 154 (97.5%) were aware about the term organ donation, and 129 (81.6%) thought it was ethically and morally correct to donate an organ.

All values stated for first years and fourth years in the subsequent tables are “participants agreed to the mentioned statement”.

### Assessment of knowledge

**Figure 1:**
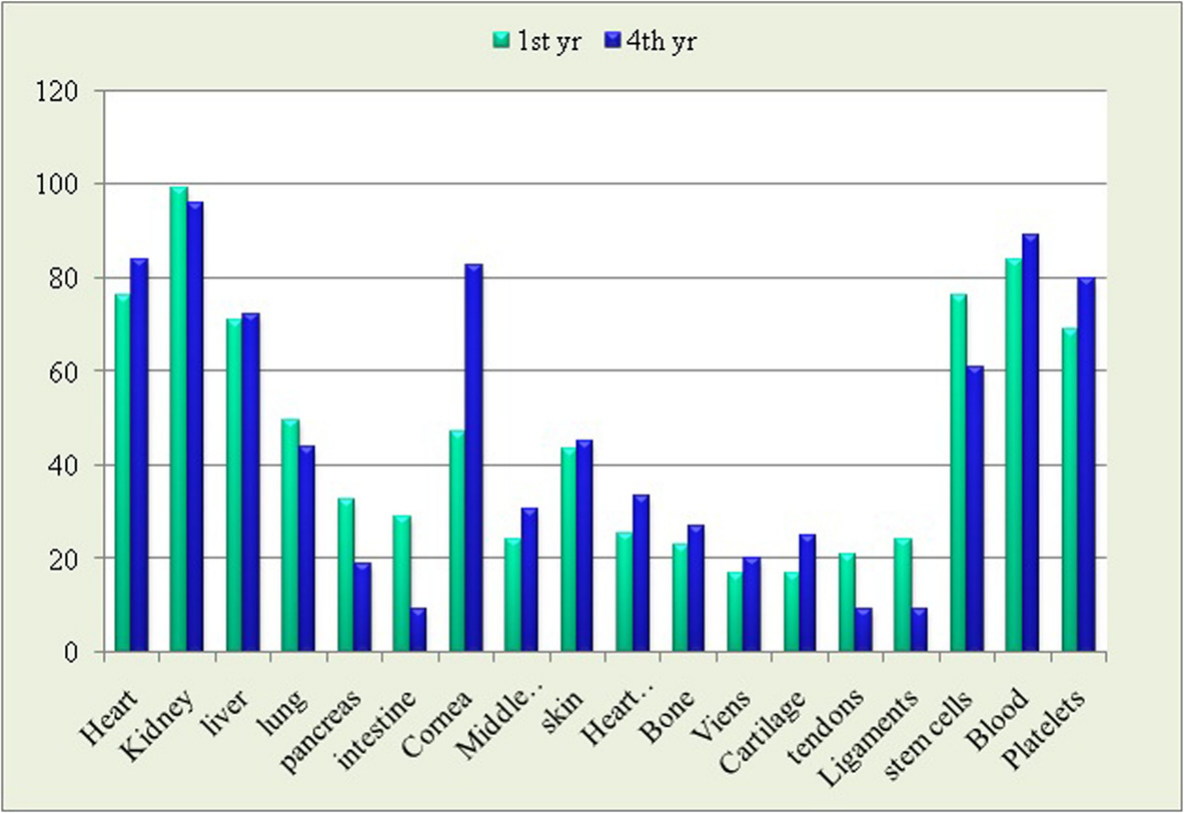
Organs that can be donated (%).

Questions were asked to determine the depth of knowledge of medical students regarding issues essential for organ procurement (Tables 1 and 2). Regarding the criteria involved in organ transplant, 153 (96.8%) was aware of the term “organ compatibility”, HLA complement system and blood grouping recorded a total of 149 (94.3%) and 114 (72.2%) affirmative respectively. Highest knowledge was regarding kidney donation followed by other tissues and organs (Figure 1). It was observed that the primary source of their knowledge was media 102(64.6%) followed by friends/family 79 (50%), Newspaper/magazines 67 (42.4%), Seminars 52 (32.9%) and only a few 44 (27.8%) reported healthcare providers.

**Table 1:** Student’s knowledge regarding important aspects of organ donation

	**All, n (%)**	**1**^**st**^**yr, n (%)**	**4**^**th**^**yr, n (%)**	**p-value**
**Is there a time duration for which organ remains viable for transplant**	141 (89.2)	69 (43.7)	72 (45.6)	0.019
**Age limit in donating organ**	101 (63.9)	62 (39.2)	39 (24.7)	0.02
**Does your religion allow you to donate an organ**	77 (48.7)	41 (25.9)	36 (22.8)	0.765
**Transplantation of Human Organ & Tissues Bill 2007**	21 (13.3)	9 (5.7)	12 (7.6)	0.359

**Table 2:** Ideal candidate for organ donation

	**All, n (%)**	**1st yr, n (%)**	**4th yr, n (%)**	
**Parents**	128 (81)	68 (43)	60 (38)	
**Siblings**	19 (12)	13 (8.2)	6 (3.8)	
**Spouse**	40 (25.3)	23 (14.6)	17 (10.8)	
**Children**	87 (55.1)	39 (24.7)	48 (30.4)	
**Friends**	32 (20.3)	20 (12.7)	12 (7.6)	
**Random**	37 (23.4)	20 (12.7)	17 (10.8)	

Only 35 (22.2%) students, 24 (15.2%) first years and 11 (7%) fourth years, were confident that they possessed enough knowledge to counsel anyone on this issue (*P=0.043*).

Majority 123 (77.8%) felt that there was a need to increase awareness regarding organ donation so more people could be encouraged to donate.

### Willingness to donate

According to their own response, 71 (44.9%) individuals of the sample population demonstrated willingness to donate their organs. Of these first years and fourth years were roughly equal with 35 (22.2%) and 36 (22.8%) students respectively. In a situation where their relative was in need of an organ 89 (56.3%) agreed to donate. When asked if donor’s family was unwilling to give consent, 84 (53.2%) stated that even then transplantation should be carried out as per the donor’s wishes. A very highly significant association (*P=0.000*) was found between willingness to donate and knowledge of allowance of organ donation in religion.

### Ethics and perceptions regarding donation

Thirty-seven (23.4%) first years selected a living healthy donor as the best option while 37 (23.4%) fourth years regarded cadavers as the best option, *P=0.045*. (Refer to Table 3).

**Table 3:** Characteristics of ideal candidate for organ donation

	**All, n (%)**	**1st yr, n (%)**	**4th yr, n (%)**
**Brain death**	28 (17.7)	10 (6.3)	18 (11.4)
**Healthy living donor**	57 (36.1)	37 (23.4)	20 (12.7)
**Paralyzed person**	1 (0.6)	1 (0.6)	0 (0)
**Cadaver (organ donation after death)**	72 (59.2)	35 (22.2)	37 (37)

Twenty-two (13.9%) first years and fourth years respectively (*P=0.024*) felt that the donor’s body is mutilated while organ harvesting. Fifty-one (32.3%) individuals felt that living donor and recipient interaction was necessary.

Determinants of recipients of organ donation yielded a variety of results. Different scenarios were stated and student response recorded (refer to Table 4).

**Table 4:** Preference for the recipient of an organ

	**All, n (%)**	**1st yr, n (%)**	**4th yr, n (%)**
**People who have never had a transplant be given priority over those who already have had one**	83 (53.8)	39 (24.7)	46 (29.1)
**Those who damaged their organ due to ill habits**	26 (16.5)	13 (8.2)	13 (8.2)
**Young patient over an elderly person**	96 (60.8)	47 (29.7)	49 (31)
**Non affording patients**	32 (20.3)	16 (10.1)	16 (10.1)
**Incentives given to donors**	75 (47.5)	42 (26.6)	33 (20.9)

Regarding their attitude to whether organ donation should be mandatory by law or only through personal choice, a high majority 127 (80.4%) were in favor of the latter with only 24 (15.2%) favoring the former.

## Discussion

Pakistan may be considered in its infancy in the field of transplantation surgery. As the proportion of population faced with chronic diseases leading to end organ failure increases, so does the demand for organ transplantation. If we are to give our patients the best treatment option available then it is imperative that we understand issues related to organ donation so we may better promote and disseminate information regarding it.

One of the key issues regarding organ donation is its allowance in religion. From this point of view 48.7% of the medical students were of the opinion that religion allows organ donation. Doubts of a disparity among religious edicts on organ donation are quelled by the fact that many religious institutions around the world recognize organ donation as an act of merit [14-18].

A study in Saudi Arabia found that the Islamic view supporting concepts of transplantation provided the strongest positive influence for organ donation [19]. This view is supported by our study; a very highly significant association (P=0.000) was seen between willingness to donate and knowledge of the allowance of organ donation in religion.

Unfortunately, only 13.3% of the student population was aware of the existence of the ‘Transplantation of Human Tissues and Organ Bill’, the knowledge of which could have far reaching impact on their decision to donate.

Knowledge regarding organ donation and transplantation was comparable amongst 1st year students and 4th year students, signifying that though there may be paucity of teaching on the subject, medical curricula, clinical exposure and foreign electives may help in understanding the various aspects of transplantation [20,21]. There was 97.5% awareness about the term ‘Organ Donation’ and exposure of fourth year students to forensics in third year and ophthalmology rotation in fourth year, led to better knowledge regarding organs that can be donated and viable time for organs.

However, level of knowledge did not translate into greater self perceived confidence and competence in counseling about organ donation. Insufficient emphasis on organ donor recruitment in the curriculum, lack of exposure and understanding about the entire transplantation process, and paucity of any large-scale organ donation public awareness campaigns in the community as well as religious and legal ambiguities, add to decrease ability to address the issue with potential donors [22-25].

In contrast to a Brazilian study [26] where medical students willingness increased as their number of years in medical school increased, in our study the number for willingness to donate remained stagnant. Since no formal course exists on Organ donation and transplantation students suffer from misconceptions that remain unaddressed throughout their medical school years. As was shown in a recent voluntary educational intervention study in Germany, 42% of the students rated their attitude towards organ donation to be influenced positively by the lecture on organ donation [27] Similarly a study in Ohio stated that students with donation and transplantation training prior to or during medical school were more knowledgeable and comfortable with obtaining information and answering patients’ donation questions [28].

Even though 81.6% thought it was ethically and morally just to donate an organ only half of this number was willing to donate an organ of their own. This percentage was distributed fairly equally amongst both first years and fourth years. Religious ambiguities, fear of organs being used for the purposes of organ trafficking, further deteriorate the will to donate [29,30]. A significant association (*P=0.024*) was found regarding whether the donor’s body is mutilated while organ harvesting, suggesting a further barrier in the process of organ donation. However the will to donate increased when asked if they would donate if a close relative was in need. This increase in willingness to donate was similar to another Turkish study done on last year university students [31]. We can explain this phenomenon by the unique fabric of the Pakistani/Asian culture, which places family as the corner stone in an individual’s life.

The close family ties make an individual sensitive to the suffering of their kin and compels them to help. Knowledge of destination of organ also adds to the satisfaction that the donation has indeed been used for the right purposes, through the right channels and without any corruption [32-35].

As was noted in our study, 45% of the medical students were willing to donate an organ. This figure was surprisingly lower compared to the 62% of the general population willing to donate an organ [29]. Possible reasons for hesitancy in medical students might be procurement procedure as was illustrated by a study in United States [36], or fear of organs used for commercial use. Further research is needed to find out the cause for this discrepancy.

Our figures were consistent with those found in Hong Kong [20], however differed from those done on Medical students in Italy [37] and United States (Ohio) [28].

Whereas fourth year medical students selected cadaveric organ donation as the best option, first year medical students favored the option of a living healthy donor. This could possibly highlight that fourth year students may be more aware of the huge shortage of organs for donation, a gap which could be bridged through cadaveric organ donation [38].

A study done on medical, nursing, dentistry and health technician students at a university in Turkey showed that majority of the students (63.1%) were unaware about the organ donation process [39]. As a first step, medical curriculum should incorporate modules specifically targeted at increasing factual knowledge and addressing issues that hinder organ donation.

There was a unanimous response that more initiatives are required to disseminate information regarding organ donation. Institutions should arrange campaigns and social events to increase awareness. Media is constantly sighted as one of the main sources of information regarding organ donation [40,41] and healthcare providers as one of the least informative [42] a trend that was similar in our study.

The issue of shortage of organs for transplantation raises the concept of ‘distributive justice’-fair division of organs. Two opposing theories in this system are equal access and maximum benefit. One establishes a system devoid of any worthiness bias while the latter works on the principle of ranking transplant candidates according to how sick they are and the number of life years gained after transplant [43]. Our results show medical students favoring the maximum benefit criteria. Another study done on the general population illustrated similar results where the distribution preference was influenced by the recipient’s behavioral life style choices [44].

Not only is there a need to address this issue at an institutional level but also to develop an ethos regarding ethical principles that inform medical practitioners decision about organ donation. As physician commitment to organ donation can positively influence the opinions and decisions of their patients, leading to higher success rates for organ procurement [45,46].

Our study ventures into a field that has been scarcely researched in Pakistan. It establishes some ground work for issues medical students face regarding organ donation. Our purpose of the study was to obtain a general overview of the opinion medical students have regarding Organ Donation. We are following up with a more in depth Questionnaire that will concentrate on specifics and perceptions regarding cadaveric donation (donation after death) only.

A high response rate is attributed to the substantial interest of medical students regarding this topic and the direct distribution and prompt collection of questionnaires. We assessed first years and fourth years to see if a difference occurs post clinical exposure. However, due to the cross sectional nature of the research a casual association cannot be drawn. Sampling used was convenient sampling and this is not superior to probability sampling. Our sample size was small and highlights attitudes regarding only one private medical institute, further studies need to be conducted in other medical colleges, private and government, for a global perspective on organ donation in Pakistan.

As the questionnaires were distributed during class, it was possible that some answers were discussed before responding, leading to a possible information bias.

## Conclusion

The results of our study are evident of discrepancies between attitude and action. Increase knowledge did not correlate significantly with the will to donate. Lack of competency in counseling suggests a dire need for improvement in curricula so as to make the next generation of healthcare professionals informed advocates regarding organ donation.

## Competing interests

The authors declare that they have no competing interests.

## Authors’ contributions

NZ participated in the design of the study and performed the statistical analysis. NFA participated in the design and conception of the study and its coordination, acquisition of data, carried out statistical analysis and drafted the manuscript. AQ participated in the conception of the study and participated in the design of the study, acquisition of data and manuscript revision. BNJ participated in the design of the study, acquisition of data and statistical analysis. All authors read and approved the final manuscript.

## Authors’ information

The authors NFA, AQ, BNJ, are medical students, and carried out this research in their fourth year of medical school, under the guidance and supervision of their facilitator NZ.

## Pre-publication history

The pre-publication history for this paper can be accessed here:

http://www.biomedcentral.com/1472-6939/14/38/prepub
